# Exploiting the structure of biochemical pathways to investigate dynamical properties with neural networks for graphs

**DOI:** 10.1093/bioinformatics/btad678

**Published:** 2023-11-10

**Authors:** Michele Fontanesi, Alessio Micheli, Paolo Milazzo, Marco Podda

**Affiliations:** Department of Computer Science, University of Pisa, 56127 Pisa, Italy; Department of Computer Science, University of Pisa, 56127 Pisa, Italy; Department of Computer Science, University of Pisa, 56127 Pisa, Italy; Department of Computer Science, University of Pisa, 56127 Pisa, Italy

## Abstract

**Motivation:**

Dynamical properties of biochemical pathways (BPs) help in understanding the functioning of living cells. Their *in silico* assessment requires simulating a dynamical system with a large number of parameters such as kinetic constants and species concentrations. Such simulations are based on numerical methods that can be time-expensive for large BPs. Moreover, parameters are often unknown and need to be estimated.

**Results:**

We developed a framework for the prediction of dynamical properties of BPs directly from the structure of their graph representation. We represent BPs as Petri nets, which can be automatically generated, for instance, from standard SBML representations. The core of the framework is a neural network for graphs that extracts relevant information directly from the Petri net structure and exploits them to learn the association with the desired dynamical property. We show experimentally that the proposed approach reliably predicts a range of diverse dynamical properties (robustness, monotonicity, and sensitivity) while being faster than numerical methods at prediction time. In synergy with the neural network models, we propose a methodology based on Petri nets arc knock-out that allows the role of each molecule in the occurrence of a certain dynamical property to be better elucidated. The methodology also provides insights useful for interpreting the predictions made by the model. The results support the conjecture often considered in the context of systems biology that the BP structure plays a primary role in the assessment of its dynamical properties.

**Availability and implementation:**

https://github.com/marcopodda/petri-bio (code), https://zenodo.org/record/7610382 (data).

## 1 Introduction

Systems biology has gained increasing attention in recent years, as researchers progressively recognized the role of molecular interaction networks, commonly referred to as Biochemical Pathways (BPs), in (dis)regulating the functioning of living cells. In a BP, molecules form a dynamical system determining how their concentrations vary over time, participating in the reactions as reactants, products, promoters, or inhibitors. To represent this variety of actors and relationships, BPs can be conveniently represented as graphs. In general, graphical representations of BPs are advantageous since they emphasize interactions, allowing one to visualize the BP structure in an intuitive way. Among the several graph representations available for BPs, Petri nets (Peterson 1977) are a common choice, especially because (i) they are well formalized, (ii) they allow both molecules and reactions to be explicitly represented, and (iii) they can be automatically extracted from standard BP formats such as SBML (Hucka *et al.* 2003). In particular, an extended version of Petri nets, called Pathway Petri Nets (PPNs) (Bove *et al.* 2020) is especially suited to describe the different roles that each molecule plays in each reaction.

BPs are analyzed *in silico* with mathematical models (Liu and Thiagarajan 2012), refined over the years to make them mimic as closely as possible several biological processes of interest. One way to characterize the behavior of a BP is by assessing properties about the concentration dynamics of the species involved, such as reachability of steady states (Angeli *et al.* 2004), occurrence of oscillatory behaviors (Ballarini and Guerriero 2010), and robustness against perturbations (Kitano 2004).

By focusing on simulation methods, these properties can be assessed on deterministic models with methods based on Ordinary Differential Equations (ODEs), or on stochastic models with algorithms such as Gillespie’s one (Gillespie 1977). Even if the two approaches are meant for different types of models, in this work, we only focus on ODEs in order to construct a homogeneous dataset of simulation results. This choice implies that the approach we will propose is aimed to analyze properties of systems that are suitable to be studied with deterministic methods.

Given a BP model in terms of ODEs, the assessment of dynamical properties is achieved by applying numerical integration methods that essentially provide trajectories over time of the concentrations of the molecular species involved. While these methods are fast enough to simulate the dynamics of a BP given a single initial condition, the assessment of global dynamical properties often requires repeating simulations for an exhaustive sampling of the parameter space, and this can become computationally expensive. In addition, as these methods require a complete specification of the underlying ODEs, they are not suitable for incomplete BPs whose kinetic parameters are often unknown and difficult to estimate. The above premises motivate the need for fast, reliable, and widely applicable techniques to analyze the dynamical properties of BPs. Besides cutting down the time to analyze large BPs and assess their global dynamical properties, such techniques could enable system biologists to perform preliminary screening of large collections of BPs.

Toward these objectives, we investigate the hypothesis that the structural properties of a BP (that we represent as a PPN) correlate with its dynamical properties (Bailey 2001). Several methods have been proposed to infer knowledge on the BP dynamics from the structure of its graph representation without performing time-consuming simulations. In Craciun *et al.* (2006), Angeli *et al.* (2010), and Yordanov *et al.* (2020), approaches based on chemical reaction network theory are proposed and implemented in order to detect specific dynamical properties such as multi-stability (by applying also optimization methods) or robustness (with a sufficient condition on the graph structure). In this paper, we further investigate the hypothesis and we propose a more general approach based on state-of-the-art Machine Learning (ML) methods and, in particular, Deep Learning methods for graphs (see Bacciu *et al.* 2020, Wu *et al.* 2021 and the references therein), to predict whether a dynamical property is satisfied on a particular PPN relying only on learned structural features, namely independently from the kinetics law and disregarding quantitative parameters (kinetic constants and concentrations). The dynamical properties investigated in this work are robustness, sensitivity, and monotonicity, and are assessed with respect to an “input” and “output” molecular species. The properties of robustness (Kitano 2004) and sensitivity (Saltelli 2008, Zi 2011) capture the effects of parameter perturbations on the BP dynamics, although in different contexts, by quantifying how a change in the concentration of the input species affects the concentration of the output species at the steady state. The property of monotonicity (Angeli *et al.* 2010), instead, aims at detecting whether a perturbation applied to the initial concentration of the input species generates an equally directed change in the concentration of the output species at the steady state. Overall, our contributions are:

- We release three datasets for public use, one for each dynamical property under study. To build the datasets, we gathered a set of manually curated BPs and converted them into ODEs. Then, we identified a set of input/output molecular species within each BP, run numerical simulations, and assessed the desired properties. Ultimately, each dataset is composed of a set of triplets for each BP, consisting of the input/output molecular species involved in the assessment and a binary target indicating whether the property holds true.
- We show experimentally that the dynamical properties of BPs under study can be predicted using the BP structure in PPN form. In this respect, we developed a neural network for graphs that processes an input PPN structure and outputs a probability indicating whether a target property holds on the corresponding BP. We also establish that exploiting the PPN structure is not only sufficient but also necessary since structure-unaware baselines are unable to learn. Furthermore, we show that our method, besides being applicable even in cases where BP parameters are unknown, has faster inference times than numerical simulations, especially as the size of the BP grows.
- We present an arc knock-out method to analyze how the prediction of a dynamical property changes when one arc of the Petri net is removed. From the biological side, this could provide insights about the role of each molecule in the BP; from the ML side, it could allow explaining model predictions by identifying the parts of the PPN that most contributed to the outcome. We show through a case study that this method can be used effectively as a proxy to reason about the biological behavior of BPs.

## 2 Background

### 2.1 Dynamical properties of biological pathways

***Robustness.*** The property of robustness quantifies the ability of a system to maintain its functionalities against internal and external perturbations. Depending on the different perturbations under study, there exist several variants of robustness; one in particular, *concentration robustness* quantifies how much a perturbation applied to the initial concentration of an input species influences the steady-state concentration of an output species. In this work, we compute a variant of concentration robustness called *α*-robustness (Nasti *et al.* 2018), which measures whether the output species steady-state concentration is contained in an interval [k−α/2,k+α/2] for some k∈R, while the initial concentration of the input species is allowed to change in predefined intervals. The computation of *α*-robustness requires many simulations of the ODEs associated with the BP, by varying the initial concentration of the input species while keeping track of the output species’ steady-state concentration. The maximum and minimum concentrations of the output species at the steady state are then used to obtain *α*, the variation range over the output. A relative version *α*-robustness, which is the one adopted in this study, is given by the formula $1-\min\left(1,\frac{\bar{\alpha }}{\epsilon }\right),$ where $\epsilon ,\bar{\alpha }\in [0,1]$ are relative versions of the input and output variation ranges, respectively. Intuitively, robustness values close to 1 are associated to input/output pairs of species where varying the input concentration does not influence the output concentration.

***Sensitivity.*** Given a generic model $o=f(x_{1},x_{2},\ldots ,x_{k})$ where $x_{i},i\in \{1,\ldots ,k\}$ are *k* independent inputs and *o* is a dependent output (both also called factors), the term Sensitivity Analysis (SA) (Saltelli 2008) identifies a class of studies whose aim is to understand how a change in a particular input (or a subset thereof) determines a variation in the output. SA methods are either local (considering small variations of only one factor at a time), or global (considering large variations of multiple factors at a time); in this work, we apply a global SA method called the Morris method (Morris 1991) to understand how a species’ initial concentration influences an output species steady-state concentration. The Morris method is based on the Elementary Effect (*EE*) metric, a local SA method to measure how an output factor *o* is influenced by an input factor *x_i_* in presence of perturbations. Formally, an *EE* is defined as $EE(i)=\frac{o_{i}^{\Delta }-o}{\Delta },$ where $o_{i}^{\Delta }=f(x_{1},x_{2},\ldots ,x_{i}+\Delta ,\ldots ,x_{k})$ and Δ is a perturbation applied to a single input factor *x_i_*. In the context of BPs, *o* is the steady-state concentration of a species, while the inputs are the initial concentration values of the remaining species. To obtain a sensitivity index with the Morris method, several EEs are computed with respect to different points in input space; these points, along with an appropriate Δ, are chosen through sampling strategies. In this work, we adopt a radial sampling strategy derived from a Sobol sequence (Burhenne *et al.* 2011), as proposed in Campolongo *et al.* (2011). Finally, the mean $\mu^{*}$ of the distribution of the absolute values of the EEs (Campolongo *et al.* 2007) and the variance *σ* of the distribution of the EEs are computed; the former is interpreted as the overall influence that *x_i_* has on *o*, while the latter estimates the effect that *x_i_* has on *o* due to the interaction with other inputs $x_{j\neq i}$. Both $\mu^{*}$ and *σ* are compared to a threshold; if any of the two exceeds the threshold, the output is declared sensitive, and not sensitive otherwise. Intuitively, for a pair of input/output species, a sensitivity of 1 (resp. 0) indicates that the output is sensitive (resp. resistant) to that particular input species.

Note that, although they share a similar objective, robustness and sensitivity are computed very differently: the former quantifies the ability of the BP to maintain its “normal” behavior (characterized by a single reference dynamics) despite a nonsmall perturbation; the latter uses perturbations to evaluate the importance of each parameter in a subset of the model domain, namely (in the variant of sensitivity we considered) by assuming that other parameters could also change.

***Monotonicity.*** Monotonic influence is a dynamical property useful to understand *how* a perturbation of the input species influences the output species. An input species is considered monotonically influential with respect to an output species if every time its initial concentration is increased or decreased, the steady-state concentration of the output species increases or decreases, respectively. The property is computed using the same EEs underlying the Morris method. In fact, this procedure computes many EEs uniformly distributed around the input domain. These are not necessarily linked to the notion of sensitivity, being just indexes to understand how a variation in an input factor determines a variation over an output factor. Since monotonicity tries to understand whether or not a variation in an input species determines an equally directed change in an output species, it can be assessed by checking the sign of all the EEs evaluated on a specific input/output pair. If all the EEs have a positive sign, the input is said to have monotonic influence over the output.

### 2.2 Pathway petri nets

Petri nets are a modeling approach originally defined to study concurrent systems, later extended to other domains such as biology. Many forms of Petri net exist: we focus on a variant apt to study BPs, called Pathway Petri Nets (PPNs) (Bove *et al.* 2020). PPNs are useful for two reasons: (i) they allow representing BPs as bipartite graphs in a simple and unambiguous way, by highlighting interactions; and (ii) the system of ODEs modeling a BP can be translated into a PPN and vice-versa. Being able to explicitly represent different types of interactions (e.g. promotion and inhibition), PPNs contain more information than standard stoichiometric matrices.

A PPN is a tuple $N=(P_{N},T_{N},\tau ,R_{\mathrm{pro}},R_{\mathrm{inh}},\rho ,m_{0})$ in which: $P_{N}$ and $T_{N}$ are finite, nonempty, disjoint sets of places (species) and transitions (reactions), resp.; $\tau :((P_{N}\times T_{N})\cup (T_{N}\times P_{N}))\to N_{0}$ defines the set of directed arcs (or relations), weighted by a non-negative integer value; $R_{\mathrm{pro}},R_{\mathrm{inh}}\subseteq (P_{N}\times T_{N})$ are the sets of promotion and inhibition arcs; $\rho :T_{N}\to (M\to R_{\geq 0})$ is a function that assigns to each transition a function corresponding to the computation of a kinetic formula (often mass action) to every possible marking m∈M; a marking describes the state of the system, assigning to every species its concentration value. The initial marking is denoted as *m*_0_.

**Figure 1.**
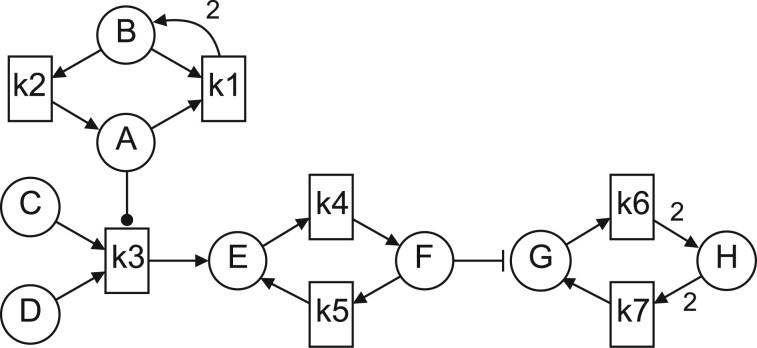
A visual representation of a Pathway Petri Net.

An example of PPN is shown in Fig. 1, highlighting its bipartite nature. Species are depicted as circular nodes, while reactions as rectangular nodes labeled by a mass action kinetic constant *k_i_*. Any arc connecting a reaction to a species indicates that the species is a product of that reaction. On the other hand, arcs connecting species to reactions are of three kinds: (i) arrow-ending, denoting reactants; (ii) dot-ending, denoting promoters; and (iii) T-ending, denoting inhibitors. Each arc is labeled with the corresponding stoichiometric coefficient that is omitted whenever it is equal to 1. Finally, the initial marking is omitted from the graphical representation and needs a separate description. This modeling approach allows transforming BPs in graphical form without losing their associated ODE semantics.

### 2.3 Neural networks for graphs

***Notation.*** For the purposes of this work, a graph is a triple 〈V,E,γ〉, where V defines its set of nodes, E⊆(V×V)={(u,v)|u,v∈V} defines its set of edges (or arcs), and γ(v)={u∈V|(u,v)∈E} is a *neighborhood function* that specifies the local connectivity of a node. We assume two functions: $\pi :V\to R^{d}$ that associates nodes to vectors of features (for some d∈N), and ω:E→A that associates edges to discrete edge labels a∈A. With a slight abuse of notation, we define the labeled neighborhood of a node γ(v,a)={u∈V|(u,v)∈E,ω((u,v))=a} as the set of neighbors of *v* with edge label *a*.

Neural networks for graphs (see Micheli 2009, Bacciu *et al.* 2020, Wu *et al.* 2021 and the references therein) are a class of ML models able to learn from graphs of arbitrary size and connectivity. To do so, they associate a vector called *node embedding* to each node. During the computation, node embeddings get simultaneously updated as a function of themselves and the neighboring node embeddings. Iterating this process ultimately allows extracting features from a graph while accounting for its structure. The basic building blocks of neural networks for graphs are Graph Convolutional Layers (GCLs). In a GCL, the embedding $h_{v}^{\ell }\in R^{d}$ (for some d∈N) of a node v∈V at layer ℓ is obtained as a function of its embedding and those of its neighbors at the previous layer ℓ−1. This concept corresponds to the following general update rule:

(1) $$h_{\gamma (v)}^{\ell -1}=\psi \left(\left\{h_{u}^{\ell -1}|u\in \gamma (v)\right\}\right)$$

(2) $$h_{v}^{\ell }=\phi \left(h_{v}^{\ell -1},h_{\gamma (v)}^{\ell -1}\right),$$

Above, ℓ∈{1,…,L} denotes the number of graph convolutional layers, *ψ* is *any* permutation-invariant aggregation function that operates on (multi-) sets of nodes, and ϕ is a function that combines the node embedding at the previous layer $h_{v}^{\ell -1}\in R^{d}$ with the aggregated embedding $h_{\gamma (v)}^{\ell -1}\in R^{d}$ representing its set of neighbors. Note that usually embeddings are initialized with node features, $h_{v}^{0}=\pi (v)$. This layered architecture allows any node embedding at layer ℓ to be updated using the information provided by nodes at a distance up to ℓ hops away. As a result, the latest layers can potentially extract global features with respect to the entire graph (Micheli 2009). When dealing with graph classification tasks, node embeddings are further aggregated to obtain layer-wise graph embeddings $h_{g}^{\ell }\in R^{d}$. The aggregation can be expressed in general as $h_{g}^{\ell }=\psi (\{h_{v}^{\ell }|v\in V\})$. In practice, a graph embedding is a single vector representation of the entire graph, which can be used by a downstream ML model for classification or regression purposes. If ϕ is differentiable and parameterized by real-valued weights, the parameters can be learned with stochastic gradient descent to minimize a given training objective.

## 3 Materials and methods

Below, we provide details about the data, the machine learning model presented, the experimental design, the evaluation protocol, and the explanatory methodology presented in this paper. In Supplementary Section A, a pictorial representation of the workflow of this study is provided for clarity.

### 3.1 Datasets

We downloaded 706 BPs from the BioModels database (Malik-Sheriff *et al.* 2019) in SBML format, discarding BPs whose specifications comprised features, such as rules and events, that were not directly translatable into PPNs. We denote the set of PPNs as $Q=\{N_{1},\ldots ,N_{m}\}$, where *m* is the number of PPNs that were successfully translated. We simulated the BPs with the *libroadrunner* library until steady-state (or until a maximum number of time units was reached) and computed the dynamical properties for all input/output species pairs of each BP, further excluding those who did not converge or generated other errors. Finally, we assembled one dataset for each of the three properties, of the form $P=\{{\left(I,O,p\right)}_{N}|N\in Q\}$, where $I\in P_{N}$ is the input molecular species, $O\in P_{N}$ is the output molecular species, and p∈{0,1} indicates whether the property holds at the steady-state (1) or not 0. Further details are provided in Supplementary Section C.

### 3.2 Graph preprocessing

The dataset entries ${\left(I,O,p\right)}_{N}\in P$ as presented in Section 3.1 were converted into training pairs by a four-step preprocessing procedure:

1. We augmented PPN *N* by adding arcs from all the reaction nodes to all their reactants to make explicit the (negative) influence of a reaction over its reactants.
2. We extracted the subgraph of *N* containing all the nodes $v\in P_{N}\cup T_{N}$ belonging to any path from *I* to *O* in the augmented graph. In case there exist no paths from *I* to *O*, the dataset entry is discarded.
3. We associated each subgraph node to a feature vector by implementing function π(v) as returning a 3D feature vector where: (i) the first entry (called *Source*) is 1 if *v* = *I*, and 0 otherwise; (ii) the second one (called *Dest*) is 1 if *v* = *O*, and 0 otherwise; (iii) the third one (called *NodeType*) is 1 if $v\in P_{N}$, and 0 if $v\in T_{N}$.
4. We associated each subgraph edge with a discrete edge label. Specifically, we implemented function ω((u,v)) as a lookup table assigning labels from the set A={reactant,promoter,inhibitor}.

A step-by-step example of preprocessing the PPN of Fig. 1 is presented in Supplementary Section B. The process resulted in a dataset of subgraphs and their associated labels $D=\{(g_{\left(I,O\right)},y_{g})|{\left(I,O,p\right)}_{N}\in P\}$, where *y_g_* = *p* for notational convenience, and $g_{\left(I,O\right)}$ are subgraphs extracted with the procedure described above. In our experiments, we only consider graphs with up to 200 nodes. Details of the graph datasets are summarized in Table 1.

**Table 1.** Summary statistics of the graph datasets used in this work.^a^

Property	No. of graphs	Positive (%)	Negative (%)	Avg. No. of nodes	Avg. No. of edges
Robustness	87 517	74 820 (85%)	12 697 (15%)	104 ± 53	199 ± 125
Sensitivity	71 896	17 074 (24%)	54 822 (76%)	100 ± 54	188 ± 127
Monotonicity	17 074	7234 (42%)	9840 (58%)	85 ± 49	151 ± 107

a We consider only graphs with at most 200 nodes as derived from the original datasets (see Section 3.1). The Positive (resp. Negative) column indicates the number and percentage of graphs for which the property holds (resp. does not hold).

### 3.3 Model

To predict the dynamical properties of BPs we propose a GCL-based architecture that takes as input a subgraph $g_{\left(I,O\right)}$ and outputs a value ${\hat{y}}_{g}$ representing the probability that the property holds on subgraph *g* with input node *I* and output node *O*. We initialize the node embeddings with their corresponding feature vector by setting $h_{v}^{0}\in R^{3}=\pi (v)$. During training, embeddings are updated as follows (for ℓ≥1):

(3) $$h_{\gamma (v,a)}^{\ell -1}=\sum_{u\in \gamma (v,a)}h_{u}^{\ell -1}$$

(4) $$h_{A,v}^{\ell }=\sum_{a\in A}E_{a}^{\ell }h_{\gamma (v,a)}^{\ell -1}$$

(5) $$h_{v}^{\ell }=\text{ReLU}\left(W^{\ell }h_{v}^{\ell }+h_{A,v}^{\ell }\right).$$

Above, $W^{\ell }\in R^{d\times d\prime }$ is a trainable weight matrix applied to the current node embedding (where d′=3 when ℓ=1 and d′=d otherwise, with *d* chosen with model selection), $E_{a}^{\ell }\in R^{d\times d\prime }$ is a trainable edge-specific weight matrix that gets applied only to neighbors of *v* with edge label *a*, and $h_{A,v}^{\ell }\in R^{d}$ is a vector that sums up the edge-specific neighbor aggregations. In practice, the contributions of neighbors of *v* are weighted separately based on their edge type [similarly to the work of Schlichtkrull *et al.* (2018), although applied in a different context]. Lastly, the embeddings are passed through a batch normalization layer (Ioffe *et al.* 2015). After the input is feed-forwarded through the *L* layers composing the network, the node embeddings are summed layer-wise to obtain *L* permutation-invariant graph embeddings, which in turn are concatenated to obtain a global graph embedding $h_{g}\in R^{dL}$:

(6) $$h_{g}^{\ell }=\frac{1}{\sqrt{\left|V\right|}}\sum_{v\in V}h_{v}^{\ell },\;h_{g}=\left[h_{g}^{1};\ldots ;h_{g}^{L}\right],$$

where [;] is vector concatenation. Finally, a probabilistic prediction from the graph embedding is obtained with:

(7) $${\hat{y}}_{g}=\text{sigmoid}\left(w^{⊺}h_{g}+b\right),$$

where $w\in R^{dL}$ is a trainable weight vector that maps the graph embedding to a scalar, b∈R is a trainable bias, and the sigmoid function computes the prediction ${\hat{y}}_{g}\in (0,1)$. The model is trained by minimizing the binary cross-entropy loss averaged over the dataset D:

(8) $$L(D)=\frac{1}{\left|D\right|}\sum_{(g,y_{g})\in D}y_{g}\;\text{log}({\hat{y}}_{g})+(1-y_{g})\;\text{log}(1-{\hat{y}}_{g}).$$

Supplementary Section C provides a visual example of how the model can process an input subgraph to produce a prediction.

### 3.4 Experimental design

Experiments are designed with two distinct goals in mind: (i) understanding whether the dynamical properties are predictable from BP structure, and (ii) understanding the impact of each model component on performances. To fulfill these objectives, we first developed a structure-agnostic baseline that simply applies a deep network to the node features, purposely ignoring node connectivity. In practice, the baseline replaced Equation 4 with $h_{A,v}^{\ell }=0$, where $0\in R^{d}$ is the 0-vector. Here, the goal was to establish whether the PPN structure is necessary for the task (Errica *et al.* 2020). We also introduce four structure-aware variants:

1. *Connectivity*: a variant where all nodes have the same feature (i.e. by setting π(v)=1,v∈V) and all edges are considered identical. This variant corresponds to replacing Equations 3, 4, and 5 with:
  (9) $$h_{v}^{\ell }=\text{ReLU}\left(W^{\ell }h_{v}^{\ell }+U^{\ell }\sum_{u\in \gamma (v)}h_{u}^{\ell }\right)$$
  where $U^{\ell }\in R^{d\times d\prime }$ is a weight matrix applied to all the neighbors.
2. *+SourceDest*: a variant similar to the above, where π(v) now returns a 2D vector containing only the *Source* and *Dest* features.
3. *+NodeType*: a variant where π(v) now returns all three features as in Section 3.2. Note that the only difference between this variant and the model presented in Section 3.3 is the neighbor aggregation, which does not take yet into account different edge types.
4. *+EdgeAware*: the variant which corresponds to the full-fledged model described in Section 3.3.

Table 2 summarizes the characteristics of the models. We remark that the purpose of this design is to isolate the contribution of a particular modeling choice with respect to the final model performance.

**Table 2.** Summary of the model variants evaluated in this work.^a^

Model	Neighbor aggregation	Input species	Output species	Node type	Edges awareness
Baseline	×	*✓*	*✓*	*✓*	×
Connectivity	*✓*	×	×	×	×
+SourceDest	*✓*	*✓*	*✓*	×	×
+NodeType	*✓*	*✓*	*✓*	*✓*	×
+EdgeAware	*✓*	*✓*	*✓*	*✓*	*✓*

a Symbols *✓* and × indicate the presence or absence of feature/modeling choices. Columns Input Species, Output Species, and Node Type refer to the three possible node features. When none is present (specifically, in the Connectivity variant), a constant feature for all graph nodes is used.

### 3.5 Evaluation protocol

We evaluated the models with stratified 5-fold Cross-Validation (CV): the datasets were split into five partitions, of which four, in turn, were used for model selection and one was used for out-of-sample evaluation. Model selection consisted of a grid search on a hold-out validation set. Precisely, we used 80% of the 4 training folds to train a model with specific hyper-parameters, and 20% to validate its performance. The hyper-parameter grid included the learning rate *η*, the node embedding size *d*, and the number of layers *L*. Among all hyper-parameter combinations, we selected those with the highest Area Under the Receiver Operating Characteristic curve (AUROC) achieved on the hold-out validation set and used it to predict the subgraphs in the test CV fold. Models were scored according to four performance metrics: AUROC, accuracy, weighted F1 (WF1), Matthews correlation coefficient (MCC), sensitivity, and specificity. For each metric, we report the average across the five hold-out folds. During training, we used a weighted sampler with replacement to even out the class imbalance of the training batches. We remark that the performances of the different models evaluated in the experiments are fairly comparable since they share the same data splits and model selection procedure while using a comparable capacity in terms of model parameters. Note also that each CV test fold is independent from the data used in training or to select hyper-parameters. Further details are in Supplementary Section D.

### 3.6 Interpretation through arc knock-out

To analyze the model predictions and to help reasoning about the role of single reactions (or products) in a BP, we introduce the following arc knock-out procedure. Given a sub-graph $\left(g,{\hat{y}}_{g}\right)$ where we omit the input and output species for readability, let us define $G=\{(g\backslash e,{\hat{y}}_{g\backslash e})|e=(u,v)\in E_{g}\}$ as the set of sub-graphs of *g* where a single edge is “knocked out”, i.e. removed without breaking connectivity. Then, given a particular edge $\bar{e}\in E_{g}$, the difference ${\hat{y}}_{g}-{\hat{y}}_{g\backslash \bar{e}}$ is the change in confidence experienced by the model as a consequence of the edge removal and can be interpreted as the contribution of arc $\bar{e}$ to the prediction ${\hat{y}}_{g}$. Furthermore, ${\hat{y}}_{g\backslash \bar{e}}$ can be compared to the property assessed on the corresponding PPN through simulations, in order to verify if the prediction is capturing the expected change in the property when a reactant-reaction (or reaction-product) interaction is absent—in other words, to understand how well the model predictions relate to the actual biology of the dynamical property. In Section 5, we present a case study based on this explanatory methodology.

## 4 Results

Table 3 summarizes the experimental results. Using AUROC as a reference metric, we see that the *+EdgeAware* variant predicts robustness and sensitivity (scoring 0.953 and 0.949, respectively). On monotonicity, we observe a minor but still positive performance (0.856), probably due to the fewer data points available for this task. The structure-agnostic baseline scores poorly on robustness and sensitivity, and random on monotonicity. In contrast, only accounting for PPN structure without using node features (the *Connectivity* variant) results in a major increase of AUROC (39% on robustness, 28% on sensitivity, and 39% for monotonicity). The implications of this result are 2-fold: on the one hand, it demonstrates that the task of predicting dynamical properties of BPs is indeed learnable; on the other hand, it shows that PPN connectivity is essential to learn the task. In this sense, we show in the Supplementary Section E that the best performances in validation are achieved whenever the model uses >6 network layers, which implies that to be proficient at this task, it is necessary to extract both local and nonlocal features from the PPNs. Another major leap in performance is obtained by the *+SourceDest* variant, where AUROC increased by 9% on robustness, by 15% on sensitivity, and by 16% on monotonicity. This shows that making the network aware of which nodes are the input and output species is beneficial for performance. Further increments (by the *+NodeType* and *+EdgeAware* variants) are minor but still positive, justifying the related modeling choices. Overall, the *+EdgeAware* variant improves the AUROC upon the baseline by 54% (robustness), 49% (sensitivity), and 63% (monotonicity). Similar improvements are achieved as regards the other evaluation metrics.

**Table 3.** Results of the evaluation.^a^

Property	Model	AUROC	Accuracy	WF1	MCC	Sensitivity	Specificity
Robustness	Baseline	0.618 ± 0.008	0.547 ± 0.182	0.586 ± 0.170	0.113 ± 0.032	0.536 ± 0.259	0.607 ± 0.273
	Connectivity	0.862 ± 0.005	0.753 ± 0.016	0.787 ± 0.012	0.423 ± 0.010	0.740 ± 0.022	0.829 ± 0.022
	+SourceDest	0.941 ± 0.002	0.903 ± 0.009	0.908 ± 0.007	0.663 ± 0.021	0.916 ± 0.012	0.824 ± 0.025
	+NodeType	0.952 ± 0.002	0.917 ± 0.007	0.921 ± 0.006	0.705 ± 0.008	0.930 ± 0.013	0.841 ± 0.032
	**+EdgeAware**	**0.953 ± 0.001**	**0.919 ± 0.006**	**0.922 ± 0.005**	**0.710 ± 0.010**	**0.930 ± 0.009**	**0.848 ± 0.014**
Sensitivity	Baseline	0.635 ± 0.006	0.424 ± 0.133	0.401 ± 0.197	0.132 ± 0.059	0.838 ± 0.155	0.295 ± 0.221
	Connectivity	0.819 ± 0.003	0.748 ± 0.014	0.762 ± 0.011	0.425 ± 0.011	0.723 ± 0.029	0.755 ± 0.026
	+SourceDest	0.936 ± 0.004	0.878 ± 0.007	0.882 ± 0.007	0.687 ± 0.014	0.831 ± 0.014	0.893 ± 0.012
	+NodeType	0.947 ± 0.003	0.893 ± 0.006	0.896 ± 0.005	0.723 ± 0.012	0.849 ± 0.017	0.907 ± 0.010
	**+EdgeAware**	**0.949 ± 0.002**	**0.896 ± 0.007**	**0.898 ± 0.006**	**0.730 ± 0.011**	**0.853 ± 0.018**	**0.910 ± 0.014**
Monotonicity	Baseline	0.525 ± 0.014	0.424 ± 0.000	0.252 ± 0.000	0.000 ± 0.000	1.000 ± 0.000	0.000 ± 0.000
	Connectivity	0.729 ± 0.003	0.654 ± 0.008	0.595 ± 0.008	0.321 ± 0.013	0.716 ± 0.025	0.608 ± 0.028
	+SourceDest	0.835 ± 0.003	0.637 ± 0.009	0.757 ± 0.009	0.511 ± 0.017	0.780 ± 0.018	0.738 ± 0.017
	+NodeType	0.838 ± 0.008	0.761 ± 0.014	0.762 ± 0.013	0.518 ± 0.023	0.763 ± 0.025	0.759 ± 0.033
	**+EdgeAware**	**0.856 ± 0.006**	**0.778 ± 0.007**	**0.778 ± 0.007**	**0.551 ± 0.015**	**0.776 ± 0.027**	**0.779 ± 0.020**

a For each metric, we report the mean and standard deviation across the five test folds. The *+EdgeAware* variant (our main proposal) is shown in bold. WF1, weighted F1; AUROC, Area Under the Receiver Operating Characteristic curve; MCC, Matthews Correlation Coefficient.

### 4.1 Time comparison: simulations and model predictions

Table 4 compares times for simulating a random sample of 30 BPs (four input/output pairs per BP) stratified by number of nodes, and to predict the same sample with the trained *+EdgeAware* model in terms. Tests were performed on a machine with 2.3 GHz Quad-Core CPU and 16 GB of RAM. Clearly, the time needed to output a prediction is orders of magnitude faster than running simulations. Moreover, such a time is relatively stable across properties (similar mean and small std), while that needed to perform simulations may vary drastically depending on the BP.

**Table 4.** Time taken (in seconds) to simulate 4 different input/output pairs in 30 different BPs, compared to the time needed to predict the same BPs with the best model.^a^

Property	Simulation			Prediction			Avg.
	Mean (SD)	Min	Max	Mean (SD)	Min	Max	Speed-up
Robustness	46.13 (134.78)	2.76	658.53	0.22 (0.03)	0.16	0.39	210×
Sensitivity	27.21 (76.15)	1.87	363.01	0.21 (0.02)	0.18	0.28	130×
Monotonicity	34.30 (81.91)	3.19	387.02	0.17 (0.02)	0.15	0.27	202×

a The column “Avg. Speed-up” is obtained as the ratio between the mean simulation time and the mean prediction time.

## 5 Case study

**Figure 2.**
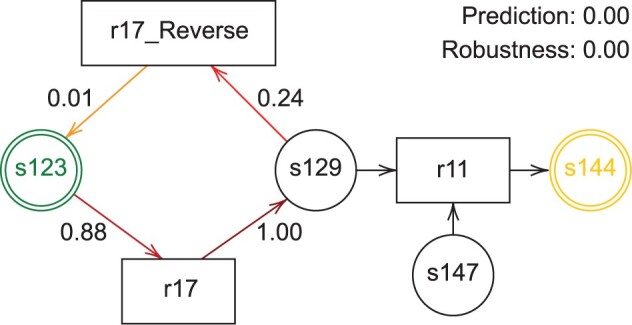
Color-coded PPN representing a subset of the EGFR signaling pathway. Input (resp. output) species is circled in green (resp. yellow). Arcs which have been removed are labelled with the corresponding prediction, where strength of increase (resp. decrease) in prediction is rendered in shades of red (resp. blue, not shown). The arcs depicted in black are not removed since their removal breaks the PPN connectivity.

We present a case study on the BP with ID BIOMD0000000394, which refers to the Epidermal Growth Factor Receptor (EGFR) signaling pathway, a well-studied BP whose alterations have been linked to cancer (Riely and Yu 2015). In particular, we study the robustness associated with input species *s123* (Gurken receptor, neuregulinAccession: P00542) and output species *s144* [Complex_br_(EGFR/./_br_GAP)]. The BP is represented by the PPN ${\left(I=s123,O=s144,p=0.19\right)}_{N_{394}}$, shown in Fig. 2. The PPN is correctly classified by the model as “not robust” with high confidence (prediction is 0). Note that the only way to produce *s144* is to have enough *s147* and *s129* to start reaction *re11*; as soon as one of those species concentrations become 0, *s144* cannot be created. Hence, the amount of *s144* will be at most equal to the minimum between *s147* and *s129*. We now describe how predictions are affected by each arc removal.

- Removing arc *(s123, r17)* makes *r17* an infinite producer of *s129*. As a consequence, the final concentration of *s144* is equal to the initial concentration of *s147*, regardless of *s123*. Thus, *s144* is expected to be robust to perturbations to *s123*. Indeed, the model correctly shifts confidence from 0 to 0.88. Accordingly, the actual robustness is 1.
- Removing arc *(r17, s129)* makes *r17* a reaction whose only objective is to destroy *s123* molecules. The initial concentration of *s123* has no way to interfere with the concentration of *s129* that ultimately contributes to producing *s144*. Thus, the output species should become more robust with respect to *s123*. Indeed, the model shifts the prediction from 0 (high confidence of being not robust) to 1 (high confidence of being robust). Accordingly, the robustness assessed with the simulation is 1. In practice, the removal of (*r17, s129*) prevents the generation of any amount of *s129*, whose initial concentration equals 0. As a consequence, without reactants, reaction *re11* never takes place, leaving *s144* to the value of its initial concentration, which is 0.
- Removing arc *(s129, r17_Reverse)* makes *r17_Reverse* an infinite producer of *s123* and, indirectly, an infinite producer of *s129* through reaction *r17*. Hence, *s144* is expected to become more robust with respect to *s123* since, independently of its initial concentration, it is available in infinite quantity. The final concentration of *s144* will be always equal to the concentration of *s147*. In accordance with the intuition, the model confidence that the PPN is robust grows from 0 to 0.24. Here, the simulations reveal that the actual robustness is 1.
- Removing arc *(r17_Reverse, s123)* makes *r17_Reverse* an infinite consumer of *s129*. Hence, only a fraction of the total amount of molecules inside the cycle (*s129, r17_Reverse, s123, r17*) will contribute to the production of *s144*. Since this amount depends on the initial concentration of *s123*, it seems reasonable to predict that the removal of the arc makes the PPN not robust. In this case, the model shifts its confidence by a mere 0.01, still predicting not robustness with high confidence. In this case, simulations resulted in a robustness value of 0.47 due to information not available to the model. Indeed, *r17_Reverse* is a fast reaction that significantly reduces the amount of *s129*, which therefore do not contribute enough to produce *s144*.

An additional case study is in Supplementary Section F.

## 6 Conclusions

In this work, we linked the fields of systems biology and ML by introducing a new approach to study BP dynamics. This work has presented three main contributions. Firstly, we released for public use three novel datasets by annotating a set of curated BPs with three dynamical properties (robustness, sensitivity, and monotonicity) assessed with high-quality numerical simulations. These datasets can be crucial to the development of novel prediction methods for BP dynamical properties. Secondly, we developed a framework that applies graph-based neural methods to the prediction of the dynamical properties of BPs. Within this framework, we have presented results that support the hypothesis that the structural properties of BPs correlate with their dynamical properties and we have shown that neural networks for graphs learn this relationship. We have also demonstrated that leveraging the BP structure is necessary to build effective predictors. Our approach is appealing because: (i) it provides a faster and yet reliable alternative to numerical-based approaches, and (ii) it works also when a complete specification of the BP parameters is unavailable, i.e. even in cases in which ODEs cannot be applied. For each property, we trained our framework on a wide range of input-output pairs of species. This allows users to study properties of *any* input-output pair of interest of a pathway of interest, or of *all* possible such pairs. Consequently, our method is suitable to speed up the analysis of BPs as well as to predict dynamical properties on large sets of BPs and input-output pairs for screening purposes. Thirdly, we introduced a method to analyze predictions by selectively knocking out arcs of PPNs. In a real-world case study, we have revealed that the behavior of the ML model when an arc is removed reflects the behavior of the BP when the corresponding chemical relation is altered. This method can be used to identify key parts of the BP and unveil their biological role. In summary, our contributions bring forth a completely new approach to studying BPs.

Our approach also has limitations. We point out at least two: (i) the learning process is affected by the quality of the simulations used to compute the dataset labels, even though this does not limit the model usability once trained; and (ii) it might not be ideal for precise property estimation, in which case numerical simulations are still preferable.

To conclude, we suggest interesting follow-ups to this work. Firstly, since in principle any ML method that computes a graph embedding fits our framework, it would be interesting to explore whether using different graph models or integrating additional information such as kinetic constants into the PPNs could improve performances. Secondly, given the broad applicability of our method, we could extend its application to other dynamical properties. Lastly, it would be useful to exploit alternative graph explainers such as GNNExplainer (Ying *et al.* 2019), or global explainability methods such as XGNN (Yuan *et al.* 2020) to complement the knowledge obtained with the proposed knock-out method.

## Author contributions

Michele Fontanesi (Data curation [equal], Software [equal], Writing—original draft [equal], Writing—review & editing [equal]), Alessio Micheli (Conceptualization [equal], Funding acquisition [equal], Methodology [equal], Supervision [equal], Validation [equal], Writing—original draft [equal], Writing—review & editing [equal]), Paolo Milazzo (Conceptualization [equal], Data curation [equal], Funding acquisition [equal], Methodology [equal], Supervision [equal], Validation [equal], Writing—original draft [equal], Writing—review & editing [equal]), and Marco Podda (Methodology [equal], Software [equal], Validation [equal], Writing—original draft [equal], Writing—review & editing [equal])

## Supplementary Material

btad678_Supplementary_Data

## Data Availability

The data underlying this article are available in Zenodo, at https://doi.org/10.5281/zenodo.7610382.
