# Supplementary material for: Exploiting the structure of biochemical pathways to investigate dynamical properties with neural networks for graphs
Source: Bioinformatics. 2023 Nov 10;39(11):btad678. doi: 10.1093/bioinformatics/btad678 (PMC10651430; doi:10.1093/bioinformatics/btad678)
Supplement: btad678_Supplementary_Data [file btad678_supplementary_data.pdf]

Supplementary material
"Exploiting the structure of biochemical pathways to investigate dynamical properties with neural networks for graphs"

A Workflow

Figure S1 shows the steps that were adopted in this study to build the property predictors.

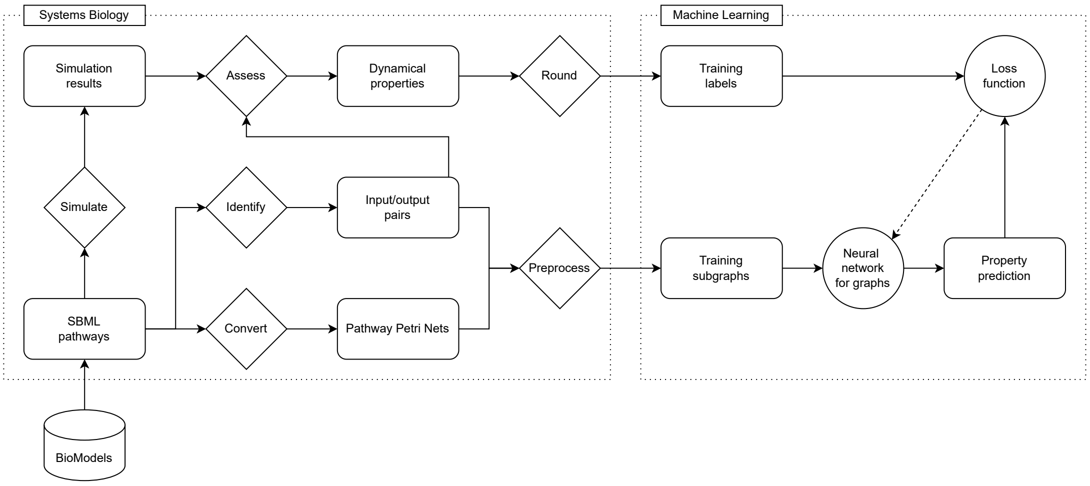

Figure S1: Workflow of this study.

We started by collecting pathway models in SBML format ("SBML pathways") from the BioModels database ("BioModels"). The pathways were simulated by varying the parameters contained in the SBML files until steady state ("Simulation results"). We identified, for each pathway in SBML format, a set of input/output molecular species pairs suitable for property assessment ("Input/output pairs"). We used the simulations and the input/output pairs to determine the dynamical properties for each (pathway, input species, output species) triplet ("Dynamical properties"). We rounded the dynamical properties at threshold 0.5 to obtain binary values indicating whether the property holds true or not for each given triplet. These became our training labels ("Training labels"). At the same time, we converted the SBML pathways into Pathway Petri Nets (PPNs) For all triples (pathway, input species, output species), we extracted the corresponding PPNs ("Pathway Petri nets", see [BM+20] for a formalization). We preprocessed the PPNs and the identified input/output pairs together to obtain a set of PPN subgraphs, each containing the input species, the output species, and all the species and reactions that influence their dynamics, obtaining a set of training subgraphs ("Training subgraphs"). We trained a neural network for graphs using the training subgraphs as input. The prediction from the network ("Predicted property") was compared to the actual training labels through a loss function, which was minimized (dashed arrow going from "Loss function" to

"Neural network for graphs") in order to learn the association between the input subgraphs and the targets.

B Simulation details

We give the details of the ODE-based simulations executed for the construction of the three datasets about the dynamical properties of robustness, sensitivity, and monotonicity, respectively. Pathways in SBML format that cannot be translated into PPNs have been discarded, typically because of the presence of species governed by SBML assignment rules rather than reactions. Moreover, pathways leading to simulation errors have been discarded as well. Since robustness and sensitivity required different simulations to be performed for the same pathways (with different initial conditions), it happened that some pathways have given valid simulation results about one property and have been discarded for the other. Part of the simulations performed for sensitivity evaluations have been also used to assess monotonicity. The number of extracted samples and pathways covered for each property are reported in Table S1, at the end of this section.

B.1 Robustness

To compute the robustness of each pathway, we proceeded as follows. First, we transformed the BP (in SBML format) into a PPN and identified all processable input/output pairs of molecular species. For each pair, we performed 100 simulations by varying the input species concentration uniformly in the interval $[-20\%, +20\%]$, which corresponds to the relative input variation range $\epsilon = 0.4$. Simulations were performed until a steady state was reached or up until 20000 time units[1]. If all simulations completed successfully, we computed the relative α-robustness value on the final state reached.

B.2 Sensitivity

For each BP, input/output pairs were identified, and simulations were performed over 32 radial trajectories. These were created using a shift d in the Sobol sequence of 11 positions and applying a remapping to the coordinates of the points in the unit hypercube to the range $[-20\%, +20\%]$ for each concentration of the input species. Simulations were performed until a steady state was reached or up until 20000-time units. Then, EEs for each input/output pair of species were computed using the following formula:

$$EE(i) = \frac{x_i}{o}\frac{o_i^{\Delta} - o}{\Delta} \tag{1}$$

Notice that this equation normalizes both the input perturbation Δ and the output response range $o_i^{\Delta} - o$. This is achieved by dividing the first one by the initial input concentration at the base point x_i, and the second one by the output concentration reached at the steady state of the base point simulation o. This normalization allowed a unique threshold to be obtained, valid across all pathways, for the values of μ^* and σ. The threshold has been set to 1/20, which corresponds to imposing that to declare a pair sensitive, an input species with a percentage perturbation of 20% should at least generate a perturbation of 1% over the concentration of the output species at the steady state. The use of a unique threshold has ultimately allowed the generation of a coherent

[1] In this case, we refer to the unit of time that characterize each BPs and defines the time scale over which the dynamical evolution of the system is studied.

dataset characterized by a well-defined set of targets, all of which are obtained with the same process and for this reason comparable. Finally, in order to get good approximations of the μ^* and σ descriptors, we took into account only input/output pairs for which the computation of at least 20 EEs was possible.

B.3 Monotonicity

To construct the dataset of monotonicity only the input/output pairs declared sensitive have been taken into account. In particular, after a specific pair was declared sensitive, the signs of all its elementary effects have been checked to be equal in order to understand whether or not the input was monotonically influencing the output.

Property	Robustness	Sensitivity	Monotonicity
Total number of samples	339062	132637	29471
Number of pathways covered	483	376	316

Table S1: Statistics of the generated datasets

C PPN Preprocessing

Here, we detail how a PPN is transformed into a subgraph that is usable by the proposed model. We will use the PPN in Figure 1, in which we have arbitrarily fixed species C as input and species F as output. For the reader's convenience, the exemplary PPN is reported again in Figure S2.

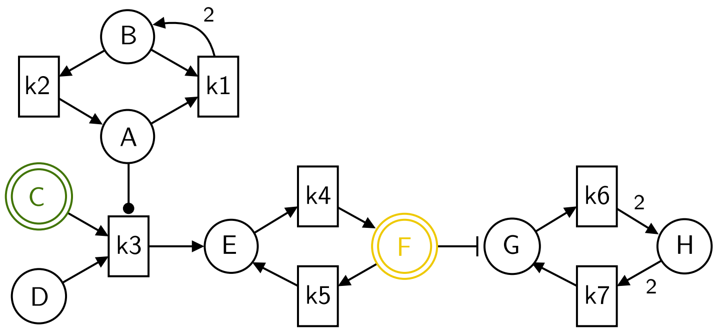

Figure S2: The PPN of Figure 1. In green: input species; in yellow: output species.

To start off, we strip out all the unnecessary information for the preprocessing, such as species and reaction names, as well as arc multiplicities, resulting in the PPN shown in Figure S3.
Subsequently (step 1. in Section 3.2), we augment the PPN by adding fictitious arcs from each reaction nodes to their reactants – species which connect to the reaction through arrowed arcs – as shown in Figure S4. This augmentation reflects the (possibly negative) influence a reaction exerts over its reactants.

Next (step 2. in Section 3.2), we isolate from the PPN the subgraph which contains the input and output species, as well as all the other nodes that are reachable from them through a directed path in the augmented graph to focus on the meaningful section that models the interaction between the

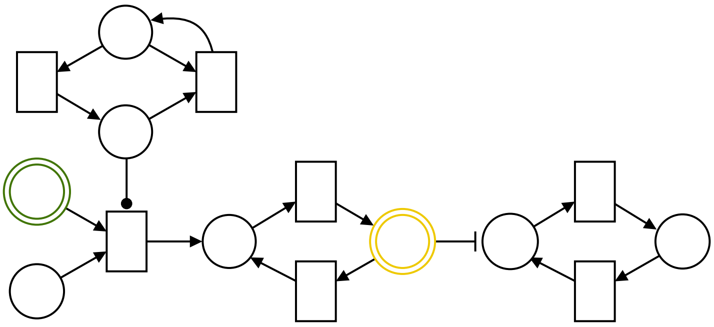

Figure S3: The PPN of Figure S2 with unnecessary information removed.

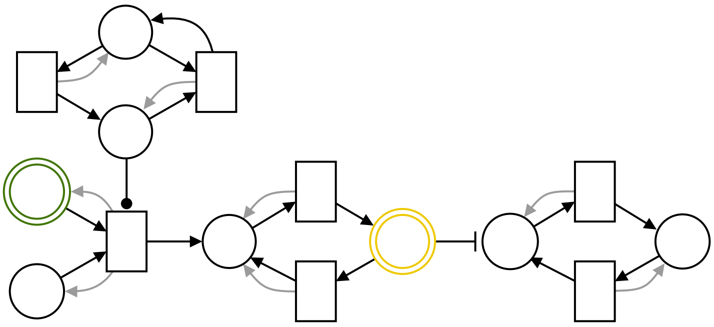

Figure S4: The augmented PPN of Figure S3. The augmented arcs are shown in gray.

analyzed species. For the particular PPN in example, this results in removing the upper subgraph and the rightmost subgraph, which are both not involved in any path from the input to the output species. The resulting subgraph is shown in Figure S5.

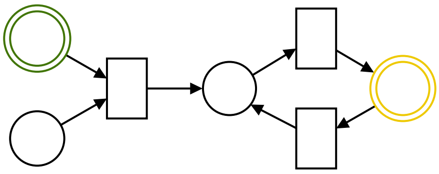

Figure S5: The subgraph extracted from the PPN of Figure S3.

Finally, we apply functions π and ω to the nodes and edges of the subgraph and remove edge directions (steps 3. and 4. in Section 3.2). The resulting undirected subgraph is shown in Figure S6. For example, node $v = 1$ is assigned a feature vector $\pi(1) = \boldsymbol{h}_1^0 =$ `[1 0 1]` because it represents the input species (first feature is 1), it does not represent the output species (second feature is 0), and it is a species (third feature is 1). Similarly, node $v = 3$ is assigned a feature vector $\pi(3) = \boldsymbol{h}_3^0$ $=$ `[0 0 0]` because it does not represent the input species (first feature is 0), it does not represent the output species (second feature is 0), and it is a reaction (third feature is 0). Label `R` is assigned to all the edges since they all are connecting a reaction to its reactants/products (i.e., they are arrow-ending). Inhibiting and promoting edges (dot-ending and T-ending, respectively) would be

associated to different labels, namely P and I.

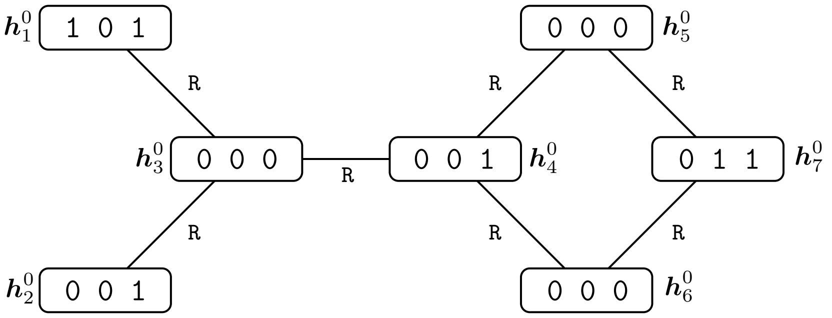

Figure S6: The subgraph derived from the PPN of S3 which is given as input to the model.

D Subgraph processing with Neural Networks for graphs

Here we detail how a subgraph, constructed as explained in Section C, is processed by the neural network for graphs. To start off, let us take the subgraph in Figure S6 as reference, which is represented by node set $\mathcal{V} = \{1, 2, 3, 4, 5, 6, 7\}$ and edge set $\mathcal{E} = \{(1, 3), (2, 3), (3, 4), (4, 5), (4, 6), (5, 7), (6, 7)\}$. Recall that the set of possible edge labels is $\mathcal{A} = \{\texttt{R}, \texttt{P}, \texttt{I}\}$, where for conciseness we abbreviate the label "reactant" with R, "promoter" with P and "inhibitor" with I. To explain the process, we focus on node $v = 3$ at layer $\ell = 1$. The neighborhood function γ (defined in Section 2.3) applied to node 3 evaluates as follows:

$$\gamma(v = 3, a = \texttt{R}) = \{1, 2, 4\}$$
$$\gamma(v = 3, a = \texttt{P}) = \emptyset$$
$$\gamma(v = 3, a = \texttt{I}) = \emptyset.$$

In this example, the objective is to calculate the embedding $\boldsymbol{h}_{v=3}^{\ell=1}$, that is, the embedding of node $v = 3$ at the first layer $\ell = 1$ (from now on, we will omit the subscript v and the superscript ℓ for conciseness). The machinery that performs a single node embedding update is termed in the article Graph Convolutional Layer (GCL). At a high level, the operations that a GCL carries out on a single node are essentially two:

- *neighboring nodes aggregation*: the embeddings of the neighbors of the node are aggregated in a single vector (Eq. (1) in the article);
- *node update*: the node embedding is updated as a function of the current node embedding and the neighborhood embedding (Eq. (2) in the article).

In our specific implementation, the neighborhood embedding $\boldsymbol{h}_{\mathcal{A},3}^1$ is calculated as in Eq. (4) in the article. Specifically, the following quantities are first computed as in Eq. (3) in the article:

$$\boldsymbol{h}_{\gamma(3,\texttt{R})}^0 = \boldsymbol{h}_1^0 + \boldsymbol{h}_2^0 + \boldsymbol{h}_4^0$$
$$\boldsymbol{h}_{\gamma(3,\texttt{P})}^0 = \mathbf{0}$$
$$\boldsymbol{h}_{\gamma(3,\texttt{I})}^0 = \mathbf{0},$$

which corresponds to summing up all the neighbors having a certain arc label separately. Notice that the sum operator is what is termed ψ in Eq. (1) in the article. In the example, all neighbors of node 3 have arc label R so the embeddings related to the other arc labels are set to be zero vectors. Next, the whole neighborhood embedding is computed as follows (Eq. (4) in the article):

$$\begin{aligned}\boldsymbol{h}^1_{\mathcal{A},3} &= \boldsymbol{E}^1_{\mathtt{R}}\boldsymbol{h}^0_{\gamma(3,\mathtt{R})} + \boldsymbol{E}^1_{\mathtt{P}}\boldsymbol{h}^0_{\gamma(3,\mathtt{P})} + \boldsymbol{E}^1_{\mathtt{I}}\boldsymbol{h}^0_{\gamma(3,\mathtt{I})} \\ &= \boldsymbol{E}^1_{\mathtt{R}}\boldsymbol{h}^0_{\gamma(3,\mathtt{R})} + \mathbf{0} \qquad\qquad + \mathbf{0} \\ &= \boldsymbol{E}^1_{\mathtt{R}}\boldsymbol{h}^0_{\gamma(3,\mathtt{R})}\end{aligned}$$

This operation corresponds to multiplying the embeddings obtained in the previous step with a dedicated weight matrix. Note that vectors $\boldsymbol{h}^0$ are $\mathbb{R}^3$ vectors, while $\boldsymbol{E}$ are $\mathbb{R}^{d\times 3}$ trainable matrices. Thus, the matrix-vector products $\boldsymbol{E}^1\boldsymbol{h}^0$ will produce $\mathbb{R}^d$ vectors, where d is the desired embedding dimension (in the experiments, d is chosen with model selection). This concludes the neighboring nodes embedding calculation.

In the next phase, the node embedding is updated according to Eq. (5) in the article:

$$\boldsymbol{h}^1_3 = \mathrm{ReLU}\left(\boldsymbol{W}^1\boldsymbol{h}^0_3 + \boldsymbol{h}^1_{\mathcal{A},3}\right).$$

Again, note that $\boldsymbol{W}$ is a $\mathbb{R}^{d\times 3}$ learnable matrix similarly to $\boldsymbol{E}$. Here, the current node embedding is first multiplied by the learnable weight matrix $\boldsymbol{W}$, and then combined with the neighboring nodes embedding with a weighted sum.

Lastly, the result of the sum is passed through an element-wise non-linear Rectified Linear Unit (ReLU) function, defined as: $\mathrm{ReLU}(x) = \max(0, x)$. The whole chain of operations is represented as function ϕ in Eq. (2).

The GCL processing is applied to all the graph nodes in parallel, and lastly a whole graph embedding at layer $\ell = 1$ is computed by aggregating all the node embeddings as follows (see Eq. (6), left side, in the article):

$$\boldsymbol{h}^1_g = \frac{1}{\sqrt{|\mathcal{V}|}}\left(\boldsymbol{h}^1_1 + \boldsymbol{h}^1_2 + \boldsymbol{h}^1_3 + \boldsymbol{h}^1_4 + \boldsymbol{h}^1_5 + \boldsymbol{h}^1_6 + \boldsymbol{h}^1_7\right),$$

where $1/\sqrt{|\mathcal{V}|}$ normalizes the sum such that the embeddings of graphs with different number of nodes will have similar magnitudes (we found empirically that this allows the training process to converge faster). The above process is repeated again on the next layer $\ell = 2$. This time around, another GCL will process the embeddings obtained at the current layer to compute the next update. After the embeddings are processed by a stack of L different GCLs, thus increasing the supporting context of each node embedding to an L-hop neighborhood, the resulting L graph embeddings are concatenated to form a single, larger graph embedding (Eq. (6), right side, in the article). The graph embedding is then linearly transformed into a scalar value, to which the sigmoid function is applied (Eq. (7) in the paper). This results in a value in the range $(0, 1)$ representing the probability that the property holds true for the input PPN. The whole process is displayed in Figure S7.

The network is trained by adjusting the values of the weight matrices $\boldsymbol{W}$ and $\boldsymbol{E}$ at each layer (which are initialized with random values) such that the cross-entropy loss between the predictions and the actual property is minimized (Eq. (8) in the paper). This is achieved with Stochastic Gradient Descent (SGD) [Rob51]. In this work, we adopted a specific SGD variant called AdamW [LH19].

E Model selection and evaluation

We provide additional details on the model selection and evaluation procedures adopted in the experiments.

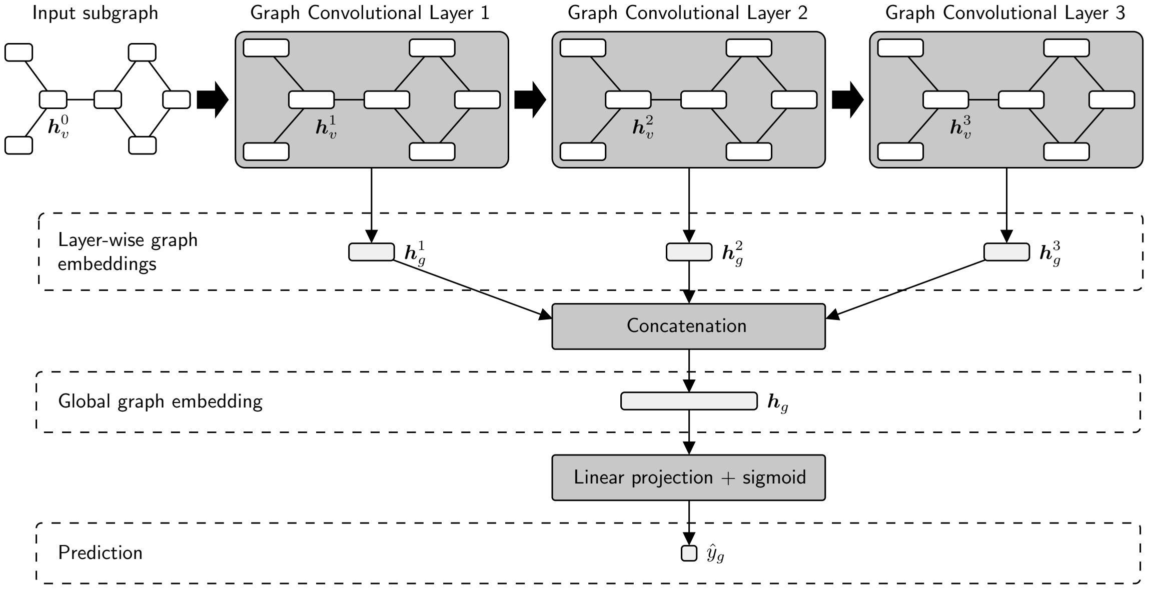

Figure S7: The whole process by which an input graph representing a PPN is processed by the model into a prediction. Here, we use $L = 3$ for simplicity; in the experiments, the optimal L is chosen through model selection. To relate each step to the article, note that node embeddings (e.g. $\boldsymbol{h}_v^1$) are computed using Eq. (5); layer-wise graph embeddings (e.g. $\boldsymbol{h}_g^1$) are computed using Eq. (6), right; the global graph embedding $\boldsymbol{h}_g$ is computed using Eq. (6), left; the final prediction $\hat{y}_g$ is computed using Eq. (7).

E.1 Hardware and Software

All experiments have been performed on a machine with 4 NVIDIA A100 GPUs and 128 GB of RAM, running on a Linux operating system. For the implementation, we used the Python programming language, and in particular the libraries `pytorch-lightning` for handling the training, `torch-geometric` for handling graph data, and `hydra` for configuration management.

E.2 Hyper-parameters

During model selection, we selected among all possible combinations of the following hyper-parameters in a grid search fashion:

- learning rate η, chosen in the interval [0.01,0.001,0.0005];
- number of layers L, chosen in the interval [2, 4, 6, 8, 10];
- embedding dimension d, chosen in the interval [128, 256, 512].

It follows that for each property, for each variant, and for each evaluation fold, we run 45 models, resulting in a total of 3375 experiments. We remark that given a property, all models shared the same training, validation and test splits.

E.3 Additional details on training

All models were trained for a minimum of 20 and a maximum of 50 epochs (although in practice we hit the upper limit only rarely); early stopping was used to end the training prematurely if the validation AUROC did not improve for 5 subsequent epochs. We used a batch size of 128, the AdamW optimizer [LH19], and a scheduler to decay the learning rate by a factor of 0.8 every 10 epochs.

E.4 Additional details on metrics

In this work, we used a set of different metrics to better evaluate the quality of the models. All of the following range from 0 to 1, with the exception of the Matthews Correlation Coefficient which ranges from -1 to 1:

- Area Under the ROC curve (AUROC): the ROC curve depicts how the ratio between the true positive rate and the false positive rate varies at different classification thresholds. The AUROC is the area under the ROC curve calculated with the trapezoidal method.
- Accuracy: it is defined as the sum of correct predictions divided by the total number of predictions.
- Weighted F1 (WF1): it is the harmonic mean between precision and recall.
- Matthews Correlation Coefficient (MCC): it measures the pairwise correlation between the model predictions and the ground truth.
- Sensitivity: also known as true positive rate, it is the ratio of positives among the total number of positives predicted.
- Specificity: also known as true negative rate, it is the ratio of negatives among the total number of negatives predicted.

F Additional results

Figure S8 shows how the average validation AUROC changes as a function of the number of layers. Each point of the curves is an average of the validation AUROC achieved by all the +EdgeAware variants that shared a common number of layers. The figure clearly shows that having a larger number of layers is essential to obtain high performances, and that this remains true across the different properties. Also notice that the performance starts plateauing at approximately 6 layers, indicating that going over 10 layers would not be anymore beneficial to improve performances.

G Additional case study

This case study concerns the analysis of pathway `BIOMD0000000035`, where we analyze the influence of the input species *DA* over the output species *DR* concerning the property of robustness, as shown in Figure S9. The model correctly predicts the fact that the robustness property holds in this case (predicted value: 0.91). As can be seen in the figure, arcs that are predicted to be particularly relevant for robustness are those connected to *Reaction13*. Let us consider, for example, arc *(DR, Reaction13)*: by removing it from the graph, the robustness predicted by the model decreases by 0.76 (as reported by the arc label), thus changing the assessment of this property into "non robust".

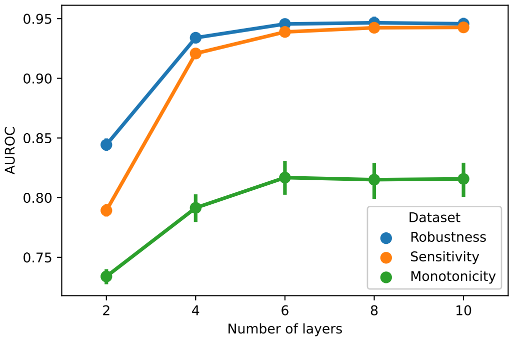

Figure S8: Influence of number of layers over the validation performance (+EdgeAware variant). Vertical bars indicate standard deviations.

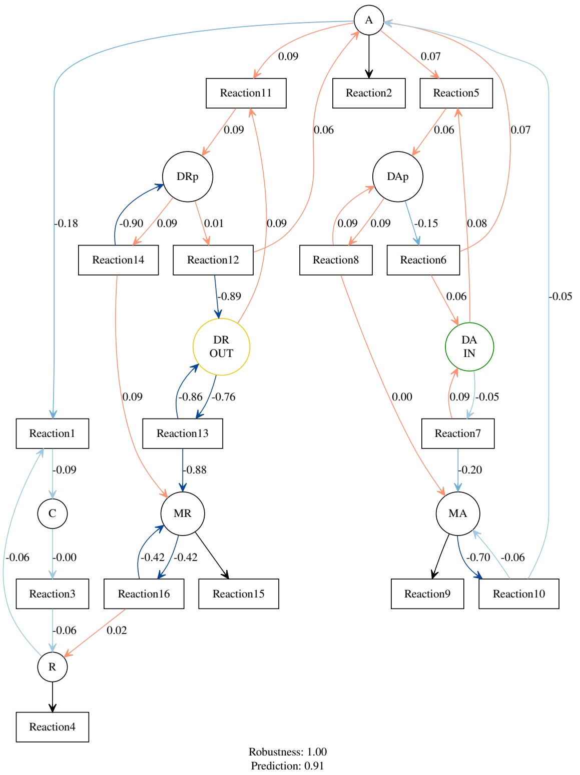

Figure S9: Knock-out graph of pathway `BIOMD0000000035` with input species DA and output species DR, where we analyze the robustness property.

Let us remark that in general, understanding the behavior of this pathway is challenging since molecules coming from DA can influence DR before reaching the arc under analysis. However, numerical simulations confirmed that after the removal the pathway should be considered not robust.

This confirms that the knock-out method has successfully predicted the consequences of the arc removal action. To give more insights on why the removal of arc *(DR, Reaction13)* decreases the robustness, it is helpful to analyze the change in the behaviour of the *DR* concentration as shown in Figure S10, that evolves from a steady oscillation to a decreasing one.

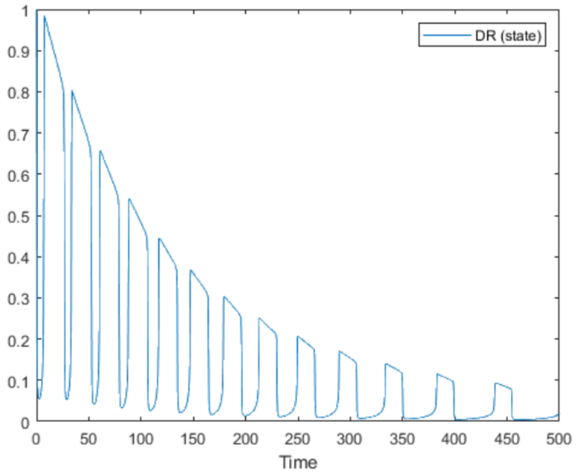

Figure S10: Behaviour of output species *DR* after the removal of arc *(DR, Reaction13)* in pathway `BIOMD0000000035` with input species *DA* and output species *DR*.

This result highlights that the model has identified a structural pattern in the PPN and used it to give insights on the BP behavior.

References

[Rob51] Herbert E. Robbins. "A Stochastic Approximation Method". In: *Annals of Mathematical Statistics* 22 (1951), pp. 400–407. URL: `https://api.semanticscholar.org/CorpusID:16945044`.

[LH19] Ilya Loshchilov and Frank Hutter. "Decoupled Weight Decay Regularization". In: *ICLR*. 2019. URL: `https://openreview.net/forum?id=Bkg6RiCqY7`.

[BM+20] Pasquale Bove, Alessio Micheli, et al. "Prediction of Dynamical Properties of Biochemical Pathways with Graph Neural Networks". In: *BIOSTEC - BIOINFORMATICS Proceedings*. INSTICC. SciTePress, 2020, pp. 32–43. ISBN: 978-989-758-398-8. DOI: `10.5220/0008964700320043`.
